# Use of the heads-up NGENUITY 3D Visualization System for vitreoretinal surgery: a retrospective evaluation of outcomes in a French tertiary center

**DOI:** 10.1038/s41598-021-88993-z

**Published:** 2021-05-11

**Authors:** Pierre Kantor, Frédéric Matonti, Fanny Varenne, Vanessa Sentis, Véronique Pagot-Mathis, Pierre Fournié, Vincent Soler

**Affiliations:** 1 Retina Unit, Ophthalmology Department, Pierre-Paul Riquet Hospital, Toulouse University Hospital (CHU Toulouse), Place Baylac, 31059 Toulouse Cedex, France; 2 Centre Monticelli Paradis, 433 bis rue Paradis, 13008 Marseille, France; 3 CNRS, Timone Neuroscience Institute, Aix-Marseille University, Marseille, France; 4 University of Toulouse III, Toulouse, France

**Keywords:** Medical research, Outcomes research, Eye diseases

## Abstract

Heads-up three-dimensional (3D) surgical visualization systems allow ophthalmic surgeons to replace surgical microscope eyepieces with high-resolution stereoscopic cameras transmitting an image to a screen. We investigated the effectiveness and safety of the heads-up NGENUITY 3D Visualization System in a retrospective evaluation of 241 consecutive vitreoretinal surgeries performed by the same surgeon using conventional microscopy (CM group) over a 1-year period versus the NGENUITY System (3D group) over a consecutive 1-year period. We included for study vitreoretinal surgeries for treatment of retinal detachment (RD) (98 surgeries), macular hole (MH) (48 surgeries), or epiretinal membrane (ERM) (95 surgeries). A total of 138 and 103 eyes were divided into 3D and CM groups, respectively. We found no differences in 3-month postoperative rates of recurrence of RD (10% versus 18%, p = 0.42), MH closure (82% versus 88%, p = 0.69), or decrease in central macular thickness of ERMs (134 ± 188 µm versus 115 ± 105 µm, p = 0.57) between the 3D and CM groups, respectively. Surgery durations and visual prognosis were also similar between both groups. We consolidate that the NGENUITY System is comparable in terms of visual and anatomical outcomes, giving it perspectives for integration into future robotized intervention.

## Introduction

The first fixed surgical microscopes arrived in the 1920s with Nylen, a Swedish ear, nose, and throat specialist, but it was not until 1946 that Perritt from Chicago made use of one for ophthalmology surgery^1–6^. Barraquer^4^ testified in 1980 that microscopes had made it possible to not only visualize previously inaccessible eye structures, but also to develop more precise surgical techniques, smaller instruments, and finer suture materials. Indeed, the advent of phacoemulsification by Kelman^7^, pars plana vitrectomy by Machemer et al.^8^, and non-perforating filtration surgery by Krasnov^9^ was during the 1960s and 1970s. The contribution of these techniques was considerable to the treatment of many diseases. Today vitrectomy is considered the reference technique for the surgical treatment of macular hole (MH) and epiretinal membrane (ERM), and it also harbors a wide range of indications for retinal detachment (RD)^10,11^.

Heads-up three-dimensional (3D) surgical visualization systems allow ophthalmic surgeons to free themselves of the eyepieces of conventional surgical microscopes, and to replace them by high-resolution dual-camera systems that retransmit an image on a screen in front of the surgeon. This switch to all-digital technology represents a major breakthrough in the conception of surgical microscopes in ophthalmology. There are now three main commercial models: the Alcon NGENUITY 3D Visualization System (Alcon Laboratories, Fort Worth, TX), the TrueVision 3D Visualization System (Leica, Wetzlar, Germany), and more recently the ARTEVO 800 system (Zeiss, Oberkochen, Germany).

Published studies have already reported on the use of these technologies in vitreoretinal surgery^12–18^, but only a few studies have included large series of patients^19,20^. The aim of this study was therefore to evaluate the effectiveness and safety of the NGENUITY System after 1 year of continuous use in our current practice by the same surgeon. We performed a retrospective evaluation of 241 consecutive vitreoretinal surgeries performed using conventional microscopy over a 1-year time period versus the NGENUITY System over a consecutive 1-year time period. We also describe the benefits and drawbacks of using the NGENUITY System according to both our experience and reports in the literature.

## Materials and methods

### Study design

**Figure 1:**
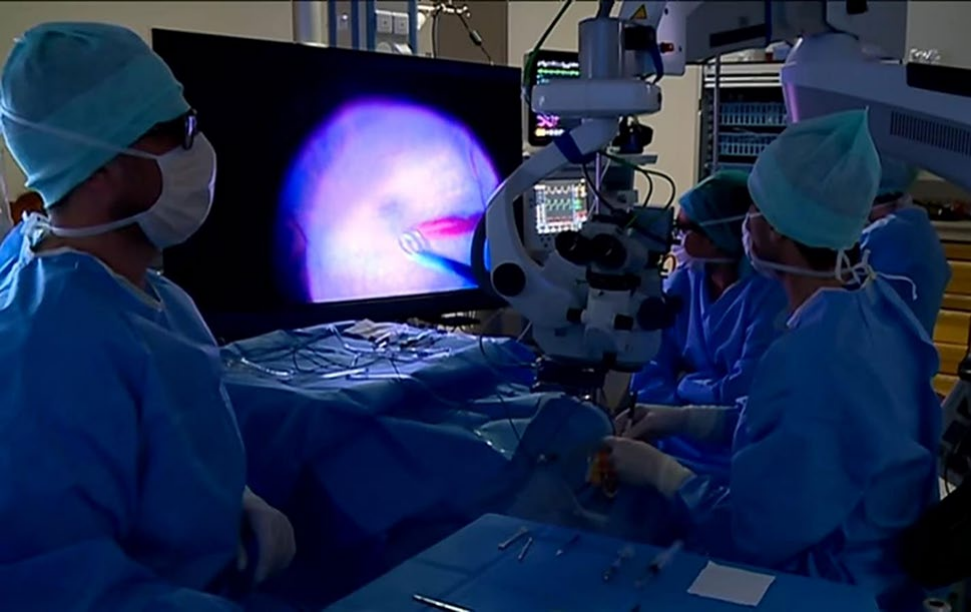
Our operating room configuration with the heads-up NGENUITY 3D Visualization System. The microscope eyepieces were left in place for the conventional microscopy (CM) group and replaced by the three dimensional (3D) camera system for the 3D group with the NGENUITY v1.2.9 software version.

**Figure 2:**
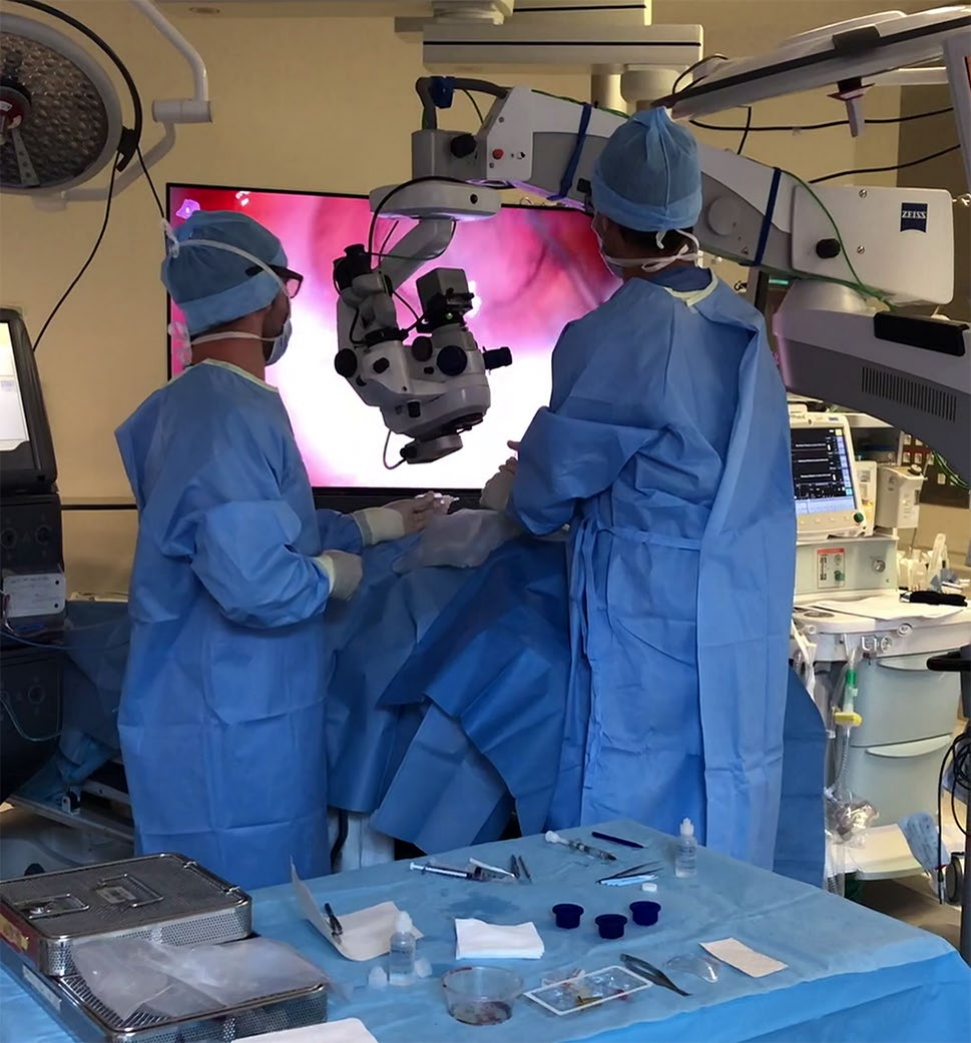
Use of the heads-up NGENUITY 3D Visualization System with a patient in semi-sitting position in our center. Patients who can only be operated on in a semi-sitting position can be operated on by the surgeon standing.

We conducted a retrospective, descriptive, comparative study in our ophthalmology department in Toulouse University Hospital (Occitanie, France) over a 2-year time period. We compared two different patient series consecutively operated on between the 29th May 2017 and 28th May 2018 or the 29th May 2018–27th May 2019 using either a conventional microscope (the CM group) or the NGENUITY System (the 3D group), respectively. Patients were thus separated into these two groups solely based on consecutive inclusion without prior comparison of demographic or anatomical characteristics. An official waiver of ethical approval was granted from the IRB of Toulouse University Hospital given the retrospective nature of the study as asserted by French Jardé law. All the procedures performed were part of routine care, and both in accordance with institutional guidelines and with the principles and regulations of the Declaration of Helsinki. Informed patient consent was obtained from participants or their relatives accordingly. The authors affirm that healthcare staff present in the images of Figs. 1 and 2 have given informed consent for publication.

### Patient selection

Patients over the age of 18 years old and having undergone vitrectomy or scleral buckling for the surgical treatment of rhegmatogenous retinal detachment, full-thickness MH, or ERM by the same experienced vitreoretinal surgeon (Vincent Soler MD, PhD) were included for study. Patients with exudative retinal detachment, tractional retinal detachments, and retinal detachment secondary to an open globe injury or secondary to MH were excluded.

### Surgical procedure

The choice of anesthesia type was left to the anesthetist’s discretion between general or locoregional peribulbar anesthesia with 10-min balloon compression. Three-port 25- or 27-gauge pars plana vitrectomy was performed with a CONSTELLATION Vision System (Alcon Laboratories, Fort Worth, TX, USA) using the manufacturer’s recommended aperture diaphragm of approximately 1/3 in order to optimize visualization while limiting retinal light exposure. Endoillumination levels were set at the beginning of the surgeries to 25% and 40% of maximum output for patients in the 3D group and CM group, respectively. These levels were adjusted when necessary to optimize retinal visualization. In the event of combined surgery, a classical phacoemulsification was performed in the first surgical step and a hydrophobic acrylic monofocal implant (ARTIS PL Cristalens, Lannion, France) was placed in the capsular bag. The peripheral retina was checked before trocar removal and sclerotomies were sutured if necessary after checking for leaks. All procedures were performed using an OPMI LUMERA 700 surgical microscope and a non-contact wide-angle RESIGHT Viewing System (Zeiss, Oberkochen, Germany). The microscope eyepieces were left in place for the CM group and replaced by the NGENUITY 3D camera system for the 3D group (refer to Fig. 1) with the NGENUITY v1.2.9 software version. The system had already been used for surgery in a test phase by the vitreoretinal surgeon on 60 patients before patient inclusion for study. We were thus able to include patients for study without the risk of learning bias as soon as the equipment was acquired. We did not use intraoperative OCT nor color filters.

### Data collection

The computerized surgical records obtained from our operating theatre management software (Centricity Opera, GE Healthcare, Chicago, USA) made it possible to recover all patient identities, as well as the surgery durations recorded by the different operating room staff present during the different surgeries. All staff received the same instructions; start the time at the start of surgery, stop the timer after dressing finalization. Details on the surgery performed, preoperative characteristics, and follow-up were collected from these computerized reports. All data has been anonymized for publication purposes.

### Evaluation of effectiveness and safety

The primary endpoints used for the analysis and comparison of effectiveness and safety were based on anatomical outcomes. Accordingly, we assessed the surgeries using conventional microscopy versus the NGENUITY System by comparing the rates of recurrence of RD, MH closure, and reduction in central macular thickness in the case of ERMs at 3 months after surgery. The secondary endpoints analyzed were surgery durations and 3-month postoperative best-corrected visual acuity (BCVA) measured in LogMAR (logarithm of the minimum angle of resolution). We analyzed patient demographic characteristics and ophthalmology history, as well as the main prognostic factors recognized in the literature for each eye disease, MH diameter, and the primary or secondary nature of ERMs in order to ensure CM-group and 3D-group comparability. Surgical techniques were also compared in order to avoid analytical biases.

### Statistical analyzes

Data were analyzed by univariate analysis by comparing the CM and 3D groups for each parameter described in the former paragraph. Analyzes were performed on all diseases combined, but also independently for each MH, RD, and ERM disease subgroup (subgroup analysis). Qualitative variables were compared using the Chi-squared test or the Fisher’s exact test when applicable. Quantitative variables were compared using the Welch’s t-test and Student’s t-test. When group numbers were too small, the non-parametric Mann–Whitney U test was used. The significance level retained was the classic 5% threshold (p < 0.05). All calculations were performed using Excel 2018.

## Results

### Description of the patient series

A total of 224 patients were included for study, divided into patients who underwent surgery with the NGENUITY System (n = 131) or with conventional microscopy (n = 96). Note that three (0.45%) patients were operated on via conventional microscopy in their first eye and then via the NGENUITY System on their contralateral eye 1 year later. A total of 138 (57%) eyes and 103 (43%) eyes in the 3D and CM groups were included for study, respectively. Patient demographic data showed no difference between 3D and CM groups in terms of age (p = 0.69), sex (p = 0.17), preoperative refraction (p = 0.35), and ophthalmology history (p = 0.084). Table 1 summarizes the demographic data and the ophthalmology history of patients included for study.

**Table 1:** Demographic data and ophthalmology history of patients included for study.

	3D group	CM group	p value
Number of patients, n	131	96	
Number of eyes, n (% total)	138 (57%)	103 (43%)	
Age in years, mean (± SD)	65.3 (± 12.7)	65.9 (± 12.8)	0.69^d^
**Sex, n (% total eyes)**			0.17^e^
Male	67 (49%)	60 (58%)	
Female	71 (51%)	43 (42%)	
**Side, n (% total eyes)**			0.9f.
Left	80 (58%)	58 (56%)	
Right	58 (42%)	45 (44%)	
**Preoperative refraction, n (% total eyes)**			0.35^f^
Low myopia^a^	66 (48%)	42 (41%)	
Low hyperopia^b^	56 (41%)	43 (42%)	
High myopia^c^	16 (12%)	18 (17%)	
**Lens status, n (% total eyes)**			0.30^e^
Phakia	86 (63%)	57 (55%)	
Pseudophakia	51 (36%)	46 (45%)	
Aphakia	1 (0.73%)	0 (0%)	
**Ophthalmology history, n (% total eyes)**			0.084^f^
No	70 (50.72%)	65 (63.14%)	
Yes			
Anterior segment surgery	5 (3.6%)	6 (5.82%)	
RD	16 (11.6%)	11 (10.68%)	
OHT/Glaucoma	10 (7.24%)	6 (5.85%)	
Macular surgery	12 (8.69%)	3 (2.91%)	
Eye diseases (RVO, ARMD, ME)	18 (13.04%)	10 (9.71)	
Posterior uveitis	9 (6.52)	2 (1.94%)	
Retinopexy	6 (4.34%)	4 (3.88%)	
Non-perforating contusion	2 (1.45%)	2 (1.94%)	

*3D group* three-dimensional group, *ARMD* age-related macular degeneration, *CM group* conventional microscopy group, *ME* macular edema, *OHT* ocular hypertension, *RD* retinal detachment, *RVO* retinal vein occlusion, *SD* standard deviation.

^a^Low myopia: spherical equivalent of 0–5.5 D.

^b^Low hyperopia: spherical equivalent of 0–4.5 D.

^c^High myopia: spherical equivalent of < − 6 D or axial length > 26 mm.

^d^Welch’s t-test.

^e^Fisher’s exact test.

^f^: Chi-squared test.

### Surgical procedures

A total of 241 vitreoretinal surgeries were performed without incident: 98 vitrectomies and scleral buckling surgeries were performed for the treatment of RD, 48 for the treatment of MH, 95 for the treatment of ERM, and 44 patients underwent combined surgery with phacoemulsification and intracapsular implantation. The 25-gauge was used for the majority of surgeries in both patient groups. Outpatient surgery was also favored in more than 80% of cases in both groups. Regarding surgery for RD and ERM, the surgical techniques did not differ between the 3D and CM groups. Internal limiting membrane peeling during surgery on patients with MH was slightly more frequent in the 3D group but was not statistically significant (p = 1). The indications for surgery and the surgical techniques performed in our study series are summarized in Table 2. We report no incidents requiring intraoperative re-installation of conventional microscope eyepieces when the NGENUITY System was being used. The vitreoretinal surgeon described a fast learning curve on his behalf for use of the NGENUITY System during the test phase described in materials and methods.

**Table 2:** Indications for surgery and the surgical techniques performed in the three-dimensional (3D) and conventional microscopy (CM) patient groups.

	3D group	CM group	p value
**Indication for surgery, n (% total eyes)**			0.25^a^
RD			
Vitrectomy	56 (41%)	35 (48%)	
Scleral buckling alone	3 (2.2%)	4 (3.9%)	
ERM	49 (36%)	46 (45%)	
MH	30 (22%)	18 (17%)	
**Combined surgery, n (% total eyes)**			0.81^b^
No	114 (83%)	83 (81%)	
Yes	24 (17%)	20 (19%)	
**Vitrectomy gauge, n (% total eyes)**			0.065^a^
25-Gauge	117 (85%)	75 (73%)	
27-Gauge	17 (12%)	24 (23%)	
Scleral buckling	3 (2.9%)	4 (4.9%)	
**Hospitalization type, n (% total eyes)**			0.36^b^
Outpatient	112 (81%)	89 (86%)	
Inpatient	26 (19%)	14 (14%)	
**RD**			
Retinopexy, n (% total RD)			0.261^a^
Cryoapplication	37 (63%)	20 (51%)	
Endolaser	18 (31%)	14 (36%)	
Cryoapplication + endolaser	2 (3.4%)	0 (0%)	
Scleral buckling, n (% total RD)			0.165^a^
No	55 (93.2%)	32 (82%)	
> 2 quadrants	2 (1.7%)	4 (10%)	
2 quadrants	1 (1.7%)	3 (7.7%)	
1 quadrant	1 (1.7%)	0 (0%)	
Internal tamponade agent, n (% total RD)			0.720^a^
C2F6	45 (76%)	30 (86%)	
Silicone oil 1000	4 (6.8%)	4 (10%)	
Oxane HD	3 (5.1%)	1 (2.6%)	
SF6	2 (3.4%)	0 (0%)	
C3F8	1 (1.7%)	0 (0%)	
Silicone oil 5000	1 (1.7%)	0 (0%)	
**MH**			
Flap , n (% total MH)			0.061^a^
Yes	17 (57%)	8 (44%)	
No	8 (27%)	10 (56%)	
Free flap	5 (17%)	0 (0%)	
ILM peeling, n (% total MH)			0.09^a^
Yes	30 (100%)	15 (78%)	
No	0 (0%)	3 (17%)	
Internal tamponade agent, n (% total MH)			0.28^b^
SF6	26 (81%)	18 (86%)	
C2F6	4 (13%)	0 (0%)	
**ERM**			
ILM peeling, n (% total ERM)			1^b^
Yes	37 (77%)	35 (76%)	
No	11 (23%)	11 (24%)	

*ERM* epiretinal membrane, *ILM* internal limiting membrane, *MH* macular hole, *RD* retinal detachment.

^a^Fisher’s exact test.

^b^Chi-squared test.

### Initial anatomical characteristics

The initial anatomical characteristics of the different eye diseases studied did not significantly differ between the patients in the 3D and CM groups (data summarized in Table 3). Concerning patients who underwent surgery for RD, 54% (n = 32) and 51% (n = 20) of patients presented with macular detachment in the 3D and CM groups, respectively (p = 0.58). A history of ipsilateral RD was found in 20% (n = 12) and 10% (n = 4) of patients in the 3D and CM groups, respectively (p = 0.3). The initial MH diameter was 360 (± 137) µm for patients in the 3D group and 384 (± 160) µm for patients in the CM group (p = 0.20). The initial central macular thickness of ERMs was 510 (± 141) µm and 486 (± 92.3) µm in the 3D and CM patient groups, respectively (p = 0.33), and the distribution between primary (39%) and secondary (39%) ERM was identical in both groups (p = 1). The other prognostic factors were the same in both groups (data summarized in Table 3).

**Table 3:** Initial anatomical characteristics of patients in the three-dimensional (3D) and conventional microscopy (CM) patient groups.

	3D group	CM group	p value
**Initial BCVA in LogMAR, mean (± SD)**			
Total	0.837 (± 0.741)	0.713 (± 0.606)	0.17^a^
RD	1.19 (± 0.887)	0.930 (± 0.564)	0.143^a^
MH	0.637 (± 0.312)	0.832 (± 0.393)	0.062^a^
ERM	0.531 (± 0.530)	0.487 (± 0.365)	0.64^a^
**RD**			
Macular status, n (% total RD)			0.58^b^
Detached	32 (54%)	20 (51%)	
Flat	22 (37%)	13 (33%)	
Pucker	5 (8.5%)	6 (15%)	
Lesion type, n (% total RD)			0.23^b^
Tear	45 (75%)	27 (69%)	
Hole	9 (15%)	11 (28%)	
PVR	3 (5%)	0 (0%)	
Not visible	3 (5%)	1 (2.6%)	
Ear size in quadrant lengths, n (% total RD)			1^b^
< 1	44 (91.7%)	34 (91.9%)	
> 1	3 (6.1%)	3 (8.1%)	
PVR, n (% total RD)			0.56^b^
Grade A	2 (3.4%)	2 (5.1%)	
Grade B	12 (20%)	8 (21%)	
Grade C1	4 (6.8%)	6 (15%)	
Grade > C1	3 (5.1%)	3 (7.7%)	
No	38 (66%)	24 (51%)	
History of ipsilateral RD, n (% total RD)			0.3^c^
Yes	12 (20%)	4 (10%)	
No	47 (80%)	35 (90%)	
Duration of evolution, n (% total RD)			0.782^c^
1–3 days	11 (19%)	6 (15%)	
4–7 days	16 (27%)	9 (23%)	
≥ 8 days	22 (37%)	18 (46%)	
Unknown	10 (17%)	6 (11%)	
**MH**			
CMT in µM, mean (± SD)	423 (± 63.4)	439 (± 78.8)	0.48^d^
MH diameter in µM, mean (± SD)	360 (± 137)	384 (± 160)	0.20^a^
Duration of evolution, n (% total MH)			0.29^b^
8 days–3 months	12 (0%)	10 (53%)	
3–6 months	8 (27%)	1 (5.6%)	
6 months–1 year	4 (13%)	2 (11%)	
Unknown	5 (17%)	6 (33%)	
**ERM**			
Etiology, n (% total ERM)			1^b^
Primary	29 (61%)	28 (61%)	
Secondary	19 (39%)	18 (39%)	
Initial macular thickness in µM, mean (± SD)	510 (± 141)	486 (± 92.3)	0.33^e^

*BCVA* best corrected visual acuity, *CMT* central macular thickness, *ERM* epiretinal membrane, *LogMAR* logarithm of the minimum angle of resolution, *MH* macular hole, *PVR* proliferative vitreoretinopathy, *RD* retinal detachment, *SD* standard deviation.

^a^Student’s t-test.

^b^Fisher’s exact test.

^c^Chi-squared test.

^d^Mann–Whitney U test.

^e^Welch’s t-test.

### Disease subgroup analysis of primary and secondary outcomes

Subgroup analysis did not identify any statistically significant differences in primary or secondary outcomes analyzed during surgery follow-up between 3D and CM groups (data summarized in Table 4). The overall rate of recurrence of RD in our study series was 12.6%, with a recurrence rate of 10% (6 eyes) in the 3D group and 18% (7 eyes) in the CM group (p = 0.42). The overall rate of MH closure at 3 months was 85%: 82% (n = 23) in the 3D group and 88% (n = 16) in the CM group (p = 0.69). ERM removal was successful in both groups. The overall reduction in central macular thickness of ERMs at 3 months after surgery was 134 µm: 154 (± 159) µm in the 3D group and 115 (± 105) µm in the CM group (p = 0.23).

**Table 4:** Anatomical outcomes according to disease subgroup in the three-dimensional (3D) and conventional microscopy (CM) patient groups.

	3D group	CM group	p value
**1-month postoperative BCVA in LogMAR, mean (± SD)**			
Total	0.494 (± 0.545)	0.487 (± 0.539)	0.096^a^
RD	0.706 (± 0.750)	0.516 (± 0.591)	0.18^a^
ERM	0.272 (± 0.200)	0.379 (± 0.358)	0.079^a^
MH	0.458 (± 0.243)	0.406 (± 0.337)	0.31^b^
**3-month postoperative BCVA in LogMAR mean (± SD)**			
Total	0.428 (± 0.531)	0.388 (± 0.461)	0.34^a^
RD	0.576 (± 0.691)	0.415 (± 0.508)	0.23^a^
ERM	0.241 (± 0.235)	0.283 (± 0.294)	0.49^a^
MH	0.431 (± 0.425)	0.443 (± 0.348)	0.086^b^
**1-month postoperative BCVA gain in LogMAR, mean (± SD)**			
Total	− 0.331 (± 0.640)	− 0.221 (± 0.461)	0.15^a^
RD	− 0.471 (± 0.823)	− 0.249 (± 0.706)	0.46^a^
ERM	− 0.268 (± 0.510)	− 0.115 (± 0.284)	0.082^a^
MH	− 0.179 (± 0.345)	− 0.426 (± 0.369)	0.048^b^
**3-month postoperative BCVA gain in LogMAR, mean (± SD)**			
Total	− 0.371 (± 0.707)	− 0.347 (± 0.522)	0.79^a^
RD	− 0.533 (± 0.721)	− 0.474 (± 0.716)	0.90^a^
ERM	− 0.286 (± 0.452)	− 0.208 (± 0.335)	0.38^a^
MH	− 0.199 (± 0.532)	− 0.398 (± 0.380)	0.19^b^
**3-month postoperative outcomes, n (% total eyes)**			0.322^c^
Cataract	21 (8.7%)	14 (5.8%)	
RD	7 (2.9%)	8 (3.31%)	
Secondary ERM	4 (1.65%)	3 (1.25)	
Post-operative macular edema	22 (9.1)	19 (7.92)	
PCO	1 (0.4%)	5 (2.1%)	
Other	8 (3.32%)	2 (0.83%)	
None	77 (32%)	55 (22.8%)	
**RD**			
Homolateral recurrence, n (% total RD)			0.42^d^
Not within 3-postoperative months	53 (90%)	32 (82%)	
Within 3-postoperative months	6 (10%)	7 (18%)	
**MH**			
3-month postoperative closure, n (% total MH)			0.69^c^
Yes	23 (82%)	16 (89%)	
No	5 (18%)	2 (11%)	
**ERM**			
3-month postoperative CMT in µM, mean (± SD)	364 (± 77.6)	379 (± 81.2)	0.46^a^
3-month postoperative CMT decrease in µM, mean (± SD)	− 154 (± 159)	− 115 (± 105)	0.23^a^

*CMT* central macular thickness, *ERM* epiretinal membrane, *LogMAR* logarithm of the minimum angle of resolution, *MH* macular hole, *PCO* posterior capsular opacification, *RD* retinal detachment, *SD* standard deviation.

^a^Welch’s t-test.

^b^Mann–Whitney U test.

^c^Fisher’s exact test.

^d^Chi-squared test.

All surgeries combined, there was a statistically significant improvement in 1-month and 3-month postoperative BCVA in both groups studied (p < 0.001, data not shown). The analysis of BCVA gains at 1 (p = 0.15) and 3 (p = 0.79) months after surgery for all surgeries combined was similar between 3D and CM groups. There were also no differences in BCVA gains between the CM and 3D groups for RD (p = 0.46/p = 0.90) and ERM (p = 0.082/p = 0.38) disease subgroups at 1 and 3 months after surgery, respectively. BCVA gains between the CM and 3D groups for patients with MH was only slightly different at 1 month (p = 0.048) but not at 3 months (p = 0.19) after surgery.

There was no difference in surgery duration between both groups: 45.4 (± 20.1) min in the 3D group and 46 (± 19.8) min in the CM group (p = 0.81) (refer to Table 5). Analysis of the different disease subgroups showed shorter surgery duration for RD in the 3D group: 47.9 (± 24.6) min versus 58.5 (± 4.24) min in the 3D and CM groups (p = 0.037), respectively. We found a shorter surgery duration for MH in the CM group: 40.8 (± 9.24) min versus 51.9 (± 18.6) min in the CM and 3D groups (p = 0.023), respectively. Surgery duration for ERM was similar between both groups (p = 0.68).

**Table 5:** Surgery duration according to disease subgroup in the three-dimensional (3D) and conventional microscopy (CM) patient groups.

Surgery duration in min, mean (± SD)	3D group	CM group	p value
Total	45.4 (± 20.1)	46.0 (± 19.8)	0.81^a^
RD	47.9 (± 24.6)	58.5 (± 4.24)	0.037^a^
ERM	38.5 (± 11.4)	37.5 (± 12.2)	0.68^a^
MH	51.9 (± 18.6)	40.8 (± 9.24)	0.023^a^

*ERM* epiretinal membrane, *MH* macular hole, *RD* retinal detachment, *SD* standard deviation.

^a^Welch’s t-test.

## Discussion

Heads-up 3D surgical visualization systems allow ophthalmic surgeons to replace conventional surgical microscope eyepieces with cameras retransmitting an image on a screen in front of them. Published studies have already reported on the use of these technologies in vitreoretinal surgery^12–18^, but only a few studies have included large series of patients^19,20^. Here, in this study we compared 1 year of continuous use of conventional microscopy versus 1 consecutive year of continuous use of the heads-up NGENUITY 3D Visualization System in our practice for a total of 241 consecutive vitreoretinal surgeries performed by the same surgeon. Overall, we did not find any significant differences in terms of visual outcomes, anatomical outcomes, or surgery durations between both techniques for the surgical treatment of RD, ERM, and MH. Furthermore, we summarize the benefits and drawbacks of using heads-up 3D visualization systems compared to conventional microscopy according to our experience and reports in the literature.

Firstly, we found no differences in anatomical outcomes and postoperative BCVA gains between patients operated on with the NGENUITY System versus conventional microscopy in our study series. These results are in agreement with other study series^12,13,15,19,20^ also demonstrating no differences in terms of safety or long-term visual prognosis when comparing the same techniques in vitrectomy surgery, and additionally for vitrectomy in the treatment of macular diseases^14,16,17^ and RD^18^. Among the aforementioned studies, Zhang et al.^20^, conducted the largest study on 23-gauge vitrectomy for the treatment of patients with vitreoretinal diseases in China. The authors found no differences in final BCVA, anatomical findings, or outcomes among patients operated on with conventional microscopy compared to the NGENUITY System. Similarly, a very recent French study on a series of 180 vitrectomy surgeries for treatment of patients with RD and MH also found no significant differences between both techniques^19^.

Regarding anatomical outcomes, the 3-month postoperative rate of recurrence of RD was 12.6% in our study series and showed no difference between the 3D and CM groups (p = 0.42). This value is similar to those described in the literature which vary between 12 and 15%^21,22^. In a recent meta-analysis, the rate of primary MH closure varied from 90 to 100%, depending on if internal limiting membrane peeling was performed^23,24^. Although we found no difference between 3D and CM groups (p = 0.69), these rates are slightly higher than those found in our study series here (85%). We can explain this difference by the fact that our study series initially included only some patients presenting with MH and high myopia (4 out of 10 non-closed MH), as well as only some patients with MH above 500 µm in diameter (3 out of 11 non-closed MH). The number of patients with MH was thus limited and made it difficult to make comparisons. In addition, the evolution of MH over time, an important prognostic factor, was unknown for a large proportion of cases (17% and 33% for the 3D and CM groups, respectively). This could be the cause of a comparison bias. Regarding ERM, Guber et al.^25^, have demonstrated a reduction in 91.9 µm in central macular thickness at 3 months after vitrectomy for treatment of patients with primary ERM. This decrease is in line with the decrease found in our study series (− 134 µm), showing again no difference between 3D and CM groups (p = 0.57), even if we did not separate primary and secondary ERM for data analysis. The collection of follow-up data was only carried out over a 3-month postoperative period in this current study due to loss of contact with a significant number of patients (patients referred to thus via their patient representatives). This allowed us to measure only the short-term effectiveness and safety without the possibility of drawing any long-term conclusions.

Likewise, surgery durations showed no differences between the 3D and CM patient groups in our study series as in accordance with previously published reports^12,20^. However, there was a difference between the MH and RD disease subgroups; surgery for RD in the 3D group was shorter, and surgery for MH in the CM group was shorter. An increase in internal limiting membrane peeling has been described by Talcott et al.^14^, but no significant differences were reported for this in other studies, for neither surgery for RD nor MH. Our overall surgery durations are slightly longer than those previously reported^20^. Moreover, our surgery durations do not include the time taken to set-up the operating room or to position the patient. On the other hand, our surgery durations were collected retrospectively by different healthcare staff present during the different surgeries. Data collection could therefore be staff-dependent, in turn contributing at least in part to the differences in our surgery durations compared to those previously reported.

Advantageous imaging benefits using a heads-up 3D visualization system over conventional microscopy have already been described. To begin, the field depth has been described as similar or superior in 3D versus conventional microscopes due to the better light sensitivity of the software and the HDR cameras; the aperture can be decreased and accordingly the field depth can be increased^12,17,26^. According to Franklin et al.^27^, the field depth is two-to-three times greater than that of the standard analogue surgical microscope if the aperture of the NGENUITY System camera is reduced to 30%, but this difference is not significant at higher zoom levels^26^. The sharpness is thus greater and the operator requires less accommodative effort, which is even more noticeable among older surgeons who no longer have a large reserve of accommodation^27^.

Secondly, the image is obtained by the fusion of two HDR cameras and then processed by algorithms that allow the detection and magnification of lower light levels than the human eye. Luminance is also improved^27^. Levels of endoillumination are therefore reduced while at the same time maintaining satisfactory visualization^13^. Indeed, low endoillumination values of 10%^15^, 3%^28^, and even 1%^29^ have been reported without loss of visual quality. This decrease therefore reduces the blinding effect of the light during surgeries with local anesthesia and reduces retinal phototoxicity, which is more frequent during posterior segment and particularly macular surgery^28^.

In relation to the facility of use of the heads-up NGENUITY 3D Visualization System, operators' opinions have been previously analyzed via satisfaction questionnaires to gather feedback from surgeons and by comparing fine surgical tasks in the operating room^13,15^. Palacios et al.^15^, showed that the heads-up NGENUITY 3D Visualization System was favored over conventional microscopy for the majority of the 14 surgeons who gave their feedback. Their reasoning owed to a better resolution, field depth, educational interest, and field of vision (results only met a statistically significant difference for educational interest). The same authors also demonstrated that the type of surgery performed most often with the 3D System was internal limiting membrane peeling^15^. These results are similar to recent reports from a team of four French surgeons who completed the same satisfactory questionnaire^19^. The authors found the main advantages to be better focusing under higher magnifications and that the light source can be kept at a greater distance from the retina (in order to limit macular phototoxicity).

These satisfactory questionnaires also showed a clear improvement in ergonomics, comfort, and a reduction in muscular pain for the users. Back and neck pain are frequently detected among ophthalmologists, and especially among surgeons. Indeed, 50.6% and 31.8% of ophthalmologists participating in a national study in the UK reported back and neck pain, respectively^30^. The consequences can be limited by correct eyepiece positioning or the use of a head-ups visualization system^31–33^. We also report here a fast learning curve for our main user of the NGENUITY System, which is in line with experiences in other study series^17^. Moreover, patient installation and surgery performance is easier with the heads-up system for patients presenting with a spinal deformity, such as kyphosis, or for patients requiring surgery in the Trendelenburg position^34^ or semi-sitting position. Figure 2 demonstrates use of the NGENUITY System in our center on a patient in semi-sitting position with the surgeon remaining standing.

There are additional pedagogical advantages to using a head-ups 3D visualization system. Firstly, all the operating room personnel present have access to the same and live surgical image, contrary to the classic configuration where only the surgeon can see a high definition and 3D image for the majority of the surgery^12^. In this light, the surgeon can teach more easily and allow trainee doctors to operate by reducing their installation time^33^. In the same way, the recording quality of the 2D or 3D videos provides a high-quality teaching aid for reviewing surgeries at distance or live. The pedagogical value of this system has also been studied in other surgical disciplines, particularly in digestive surgery and microsurgery^33,35^.

The transition from direct visualization using a surgical microscope to analogue visualization via an indirect digital system must fulfil certain technical criteria^27^. Two slightly different and shifted images are retransmitted by two HD cameras to a 4 K monitor and passive polarized glasses allow for the phenomenon of disparity for each eye^36,37^. Along with this, there has been a handful of drawbacks identified in the use of head-ups 3D visualization systems. For example, the latency time is 70–80 ms for the NGENUITY 3D Visualization System. In our practice, we did not perceive this latency time as a handicap; it has actually been reduced compared to former versions^13^. Furthermore, some surgical aids have been reported by surgeons as being less comfortable, resulting in asthenopia by the end of surgery. Underlying exophoria has also been put forward as a risk factor^38^, but our main operator here throughout this study presents with exophoria and did not encounter any problems during surgery. In addition, some anesthesia teams have conveyed a dislike to the large size of the visualization system with respect to complicated patient and monitoring access, especially during general anesthesia^39^. The operating room must therefore be of sufficient size and reorganized to avoid these difficulties^40^. Figure 1 shows the configuration of our operating room with the heads-up NGENUITY 3D Visualization System.

In addition to the significant potential in vitreoretinal surgery, heads-up 3D visualization systems are compatible with the performance of other types of ophthalmology surgery. This includes cataract surgery, with some teams showing effective results without increased risk of complications or increased surgery durations^40,41^. There also lies an interest in: (1) corneal transplantation, especially for lamellar keratoplasty given the high-zoom quality and field depth^42^, (2) strabismus surgery, given the surgeon does not require the external light as it is replaced by a HDR camera and gain adjustment^43^, and (3) glaucoma surgery, with the iStent implantation being simplified by the small camera sizes^15^. ARGUS II retinal implantation has also been carried out with this system as it renders it easy to perform sclerotomy and intracavitary placement^44^. Nevertheless, some surgeons have reported technical difficulties during anterior segment surgery and external eye surgeries, with difficulties in acquiring a clear image and instrument positioning. Likewise, these difficulties have also been evoked during scleral buckling^15,19,44^.

On the whole, changing to use of a single digital display system makes it easier to insert other multimodal imaging components and live details, such as vitrectomy settings (DATAFUSION software), and it can be used to guide incision positions, toric implantation, and rhexis size (VERION Image Guided module)^45^. Intraoperative OCT is also accessible on a single screen, without the surgeon having to look away or use only one of the two eyepieces^46^. Ultimately, this transition to a live, digital, and high-quality image is an essential step in enabling image transmission during robot-assisted remote surgery. From here, we can imagine a surgeon operating on a patient at distance without the need to travel. In the same way, due to the recent exponential development of artificial intelligence in ophthalmology, we can hypothesize the development of future applications that allow live operator assistance or even a 100% robotized intervention.

In conclusion, the heads-up NGENUITY 3D Visualization System appears comparable to conventional surgical microscopy in terms of effectiveness and safety in surgical treatment of RD, ERM, and MH. It has proven to be a valuable tool from an ergonomic and pedagogical point of view, while at the same time maintaining high image quality. In the future we can expect this system to be integrated into the framework of intraoperative multimodal imaging in ophthalmology surgery.

## Data Availability

The datasets analyzed and generated during the current study are available from the corresponding author upon reasonable request.
